# Supplementary material for: The speed of sight: Individual variation in critical flicker fusion thresholds
Source: PLoS One. 2024 Apr 1;19(4):e0298007. doi: 10.1371/journal.pone.0298007 (PMC10984398; doi:10.1371/journal.pone.0298007)
Supplement: Attachment — Submitted filename: Response to reviewers.docx [file pone.0298007.s001.docx]

**Response to reviewers**

**Original manuscript ID:** PONE-D-23-39116

**Original manuscript title:** “The speed of sight: individual variation in critical flicker fusion thresholds”

Dear editor and reviewers,

Thank you for your comments and suggestions on our manuscript, and for the opportunity to amend and resubmit our article. We have addressed each comment below, and will upload this rebuttal letter, titled ‘Response to reviewers’ along with:

- A marked up copy with “track changes,” titled ‘Revised Manuscript with Track Changes’.
- An unmarked version of the revised manuscript without “track changes”, titled ‘Manuscript’.

Sincerely,

Clinton Haarlem et al.

**Editor comments**

**Comment:** *Please ensure that your manuscript meets PLOS ONE's style requirements, including those for file naming. The PLOS ONE style templates can be found at*

[*https://journals.plos.org/plosone/s/file?id=wjVg/PLOSOne_formatting_sample_main_body.pdf*](https://journals.plos.org/plosone/s/file?id=wjVg/PLOSOne_formatting_sample_main_body.pdf) *and*

[*https://journals.plos.org/plosone/s/file?id=ba62/PLOSOne_formatting_sample_title_authors_affiliations.pdf*](https://journals.plos.org/plosone/s/file?id=ba62/PLOSOne_formatting_sample_title_authors_affiliations.pdf)

**Author’s response:** We have reviewed PLOS ONE’s style requirements using the offered information and have formatted the revised manuscript accordingly.

**Comment:** *Please review your reference list to ensure that it is complete and correct. If you have cited papers that have been retracted, please include the rationale for doing so in the manuscript text, or remove these references and replace them with relevant current references. Any changes to the reference list should be mentioned in the rebuttal letter that accompanies your revised manuscript. If you need to cite a retracted article, indicate the article’s retracted status in the References list and also include a citation and full reference for the retraction notice.*

**Author’s response:** We have reviewed and revised our reference list and ensured the information is complete and correct at the time of writing. We have added one additional reference, according to reviewer suggestions.

**Reviewer #1 comments**

**Comment:** *l. 59: may be mention: Muth T et al, 2023*

**Author’s response:** Thank you for bringing this insightful review to our attention. We have included this reference in our article.

**Comment:** *l. 99: ECTS, meaning what?*

**Author’s response:** We have included more information on participant compensation and explained the meaning of ECTS.

**Comment:** *l. 105: a photograph or a schematic with detailed description would be helpful for the less informed reader. Please, add size of the light source.*

**Author’s response:** Thank you for this suggestion. We have included a photograph of the testing device, and have expanded on the description.

**Comment:** *l. 115: Protocol. Participants were standing / sitting? Height of tubing was adjustable?*

**Author’s response:** We have provided more details on the protocol used for testing.

**Comment:** *l. 115: tests were performed in the morning or during the entire day?*

**Author’s response:** The hours during which testing was carried out is now included in the protocol.

**Comment:** *l. 150:* *QQ-plots?*

**Author’s response:** We have refrained from using this abbreviation and have used the term “quantile-quantile plots” instead. Additionally, we have made it more clear which R package was used to create these plots.

**Comment:** *l. 155: Pls, explain meaning of 0.8. Is there a graph?*

**Author’s response:** We have added more explanation on this part of the methods, and we have included an example graph to visualize the calculation of CFF thresholds with this method.

**Reviewer #2 comments**

**Comment:** *The manuscript may benefit from adding of more precise information about the hours of the study.*

**Author’s response:** We have included the hours during which testing was carried out in the methods section.

**Comment:** *I suggest adding a brief note about the "car package" from R (line 148/149), explaining that it is a set of functions and tools for regression analysis.*

**Author’s response:** We have included a note about the usage of the “car package”.

**Comment:** *I also recommend moving the description of normality in the results section to the very beginning, before the description of correlations to improve the readability of the description and justify the use of parametric tests.*

**Author’s response:** Thank you for this suggestion. We agree this improves the flow of the overall manuscript and have made the recommended adjustment.

**Comment:** *I think the data presented in Figure 1 would be more easily compared visually after using the same reference points on the x and y axes.*

*Description of Figure 3 - please standardize the lowercase and uppercase letters in the statistics given.*

**Author’s response:** We have ensured the three graphs now all have the same reference points for the x and y axes. We have also formatted the figure descriptions to match capitalization used in the figure.

**Comment:** *In the discussion section, the authors describe factors that may affect the differences in CFF thresholds. I agree that they may be related to neuroanatomical, neurophysiological and neuropsychological factors. Have previous studies compared genders or age groups in this regard? I lack such an explanation.*

**Author’s response:** Previous studies have found gender differences in areas such as temporal processing and visual motion processing. However, to the best of our knowledge, no specific physiological or neuroanatomical correlates have been identified to explain these differences. As the fields of psychophysics and sensory neuroscience are vast, we believe we would benefit greatly from more review studies investigating the causes underpinning gender and age related variation in sensory processing.

**Comment:** *It is not clear what the authors meant when they wrote about testing visual temporal resolution among humans in real-world conditions (line 292-294). I suggest adding a clarification.*

**Author’s response:** We have added two small examples of real-world scenarios where, we believe, variation in visual temporal resolution might be impactful.
